# Foreign

**Published:** 1842-01-08

**Authors:** 


### FOREIGN.

Dr. Kilgour, in his notes in the Edinburgh Medical and Surgical Journal for Oct. 1841, gives some interesting results as to the forms of fever which prevail in Great Britain;—that is, true typhus, not the sporadic typhoid fever which is common enough in the United States. The cases were noted in a tabular register, and the results given as accurately as could be done, without describing each case in its details.

I. The first question to determine is whether the cases of fever arose from infection. This could be shown positively as regards many of these cases ; there were, of course, no means of proving that infection really existed in some in which it was not apparent.

"The cases arising from infection, that is to say, brought from houses or families where fever existed, were, in the first year, 23.80 per cent.; in the second, 29.22 per cent.; and in the third, 53.37 per cent.

"I am certain, however, that these are considerably below the real numbers where the disease was produced from this cause ; for I have long observed a disposition on the part of the patients to conceal or deny their having been exposed to infection."

II. Next as to the eruption, or measle like exanthema, a constant, almost invariable symptom in the cases of true typhus, observed in different epidemics at Philadelphia from 1836 to 1841.

"The cases admitted with the exanthema, or the eruption of typhus, in the first year, were 31.21 per cent.; in the second, 33.54 per cent.; and in the third, 52.05 per cent. So that the proportion is here nearly the same as under the former head. By another table, which was kept during the year 1840, the per centage of those admitted from infection, and having also the exanthema was 36.39, and of those having exanthema, but where they had not been, to their knowledge, exposed to infection, it was 15.66.

"I am perfectly satisfied that this fever, call it by what name we will, is truly an exanthematous fever, though not observing the same periodicity or regularity in the cutaneous affection as those longer known to us as exanthematous fevers. I attempted for some time to keep tables of the form, colour, date of appearance, and of disappearance of exanthema ; but the results were so exceedingly variable, and the characters of the exanthema, except in so far as regarded colour, had so little apparent connection with the type of the fever, and so little influenced the practice, that I gave them up. A table which was kept in 1840 of the cases from infection, but not attended with exanthema, gives us 17.60 per cent.; but in almost every one of the cases where the fever was caught from those having the exanthema, the cutaneous eruption was present. Thus of the students, fever nurses, and domestic servants in the hospital, who took the fever, every one had the eruptive form. In many cases it is confined to one part of the body, and the spots may be few in number and not very distinct unless to a practised eye. That the numbers are not higher in this column of the table arises from this,—that entries were only made of those cases where it was general over the body. I need not now-a-days say it is different from petechiæ, and that the two sometimes exist together in the same case. In those cases that were to be fatal, the exanthema often became like purpura, the spots being most numerous on the lower extremities; but in size and shades of colour always distinct from true petechiæ where the latter were present.

"I have never known a second attack of exanthematous typhus in the same individual. It happened in several instances, that persons sent into the fever wards with inflammatory diseases, such as bronchitis, influenza, erysipelas, &c., were seized with exanthematous typhus, before they could be got out, or, if sent out, were returned on us after a variable time with that disease."

It appears that in cases where the disease was witnessed during its whole course, the exanthema was present. This explains the apparent absence of it, in many cases; they are observed for a short time, late or early, in the disease, or the eruption happens to be extremely pale.

III. *Mortality.* This was very uniform in the different years; in our epidemics it was more variable. Taking mild and severe forms together, Dr. Kilgour's proportion is one in 9.34.

"The deaths in the first ten months were 1 in 7.26, or 12.68 per cent.; in the second year, they were 1 in 9.51 or 10.13 per cent.; and in the third, 1 in 10.07 or 9.92 per cent., or close upon 10 per cent. This mortality, however, it must be observed, includes all deaths in my fever wards, and it embraces, therefore, those admitted moribund, as well as some cases not fever. Taking the whole deaths against the whole admissions for the three years, the proportion is 1 in 9.34; or, deducting from both admissions and deaths, those admitted moribund, of which the number was 10, and in which death took place within twenty-four hours after admission, and deducting in the same way 6 cases where death was not connected with fever, it is 1 in 10.89."

IV and V. The author concludes that general bleeding was injurious. "No line of treatment was found more injurious than loss of blood from the arm, and even in cases of local congestion it was found that the loss of blood locally was not well borne." In our experience we have not found that the local depletion was not well borne in cases of moderate severity without excessive fœtor or other signs of great alteration of the fluids, and cupping about the head, both scarified and dry, was singularly beneficial, in relieving troublesome symptoms at least.

VI. Blisters were rarely used; they are approved of by Dr. Kilgour in affections of different organs. We like them much in pneumonia attending typhus, and in the declining stage of the fever when there is still considerable head disturbance.

VII. Wine; the total quantity used was, on the average, one ounce and three-quarters for each patient; this is a small proportion. It is probably enough, if regard be had to the mortality alone; but if we wish to shorten the disease and increase the comforts of the patients during convalescence, more wine should be used. After the decline of the fever, there is usually a period of prostration before the full developement of convalescence. At this time wine is almost invariably of service, though not, it is true, absolutely necessary in most cases. In the earlier periods of the disease, wine is often of benefit. In some epidemics it is so in almost every case; in others the fever is more active, and approaches more closely to the inflammatory diseases, and wine is less frequently indicated.

Among the sequela of the fever were pain in the legs and walking; and abscesses of the limbs. A furfuraceous desquamation took place in many cases, something similar to that of measles; we have observed the same facts in the fever of Philadelphia, and in one case there was gangrene of the leg.

The cause of death is noted in a very indistinct manner. We give Dr. Kilgour's statement:—

"The cause of death, or rather the organ chiefly affected at the time of death was thus noted in 99 cases:

| Head. | Head and bowels. | Lungs. | Stomach and bowels. | Erysipelas. | Moribund. |
|---|---|---|---|---|---|
| 29 | 6 | 18 | 21 | 15 | 10 |

"Amongst those entered as affections of the head, one was a case of hydrocephalus, and another was found at the necropsy to be a tumour in the cerebellum. Of those entered as affections of the lungs, three were cases of pure pneumonia. Three children in one family, who died of typhus, were found to have a collection of pus, or empyema of one side of the chest. I believe, that in the hospital, or at their own house, five, if not six, children, the oldest not being sixteen, of this family were carried off by fever. They had come from the Highlands a short time previous, and were in extreme want. Of those entered as affections of the bowels, four had melæna stools, or passed blood from the bowels previous to death. Of the deaths from erysipelas, five arose from that disease attacking the glottis, or extending inwards to it from the face; and two died from being worn out by numerous superficial abscesses which followed the erysipelas in various parts of the body. Thirteen of the deaths are stated to have taken place during convulsions, and in most of these, the entry in regard to 'habits' is 'very intemperate.' Some of the cases put down as affections of the head would, but for the abundant exantheme, have been put down as *delirium tremens.* They were drunken dissipated characters. Some of them walked to the hospital on their own feet, and, notwithstanding the free use of wine after admission, and, in some cases, opiates, were cut off in two or three days at furthest.

"Besides the above, two females died of mortification of the vagina, extending upwards to the uterus; one of vibices and purpura on the second day, after having had an abortion; one of purpura with severe cutaneous hæmorrhage; and one of sphacelation following the application of a blister."

Probably the autopsies were not made in many cases; at least, it does not appear so from the text. It would be very interesting to know, from exact statistical documents, whether the disease of the glands of the intestine occurs in a certain proportion of cases of the true typhus of Great Britain. We have never found them in the typhus of Philadelphia. This is one of the principal distinctive characters between typhus and typhoid fever. The latter is the sporadic disease, very common in the United States, especially in the northern states, and in the hilly parts of the country. It is, of course, frequently designated typhus, but in in reality is different from the epidemic fever of British writers.

The duration of the fever is badly described; that is, the date of convalescence is not fixed; as to that of the discharge of the patient it is of little value. But sixteen cases of males, and eleven of females, were dismissed from the hospital before the twentieth day from the commencement of the attack. One half the cases of males were discharged between the twentieth and thirtieth days, but the largest number of discharges of females took place between the thirtieth and fortieth: the women seemed, therefore, to recover more slowly. The mortality among the men was, as is usual, greater than among women, owing, in part, perhaps, to the fact that more cases were brought in moribund; but a similar difference is true in most acute diseases. More cases occurred among women than men.

The causes of the fever could not be explained by atmospheric or other peculiarities, nor could we account for the Philadelphia epidemics.

The colleague of Dr. Kilgour, Dr. Dyce, agrees in general with his views, especially as to bleeding, the contagion of the disease, and the eruption similar to what occurred at Philadelphia. He thinks that occasionally a second attack takes place; this we have seen, but rarely. As regards bleeding, Dr. Dyce remarks:

"I quite agree with your remarks on bleeding. I found it rarely could be borne, certainly never to any extent; and when deemed necessary from the urgency of the local symptoms, a tedious convalescence invariably followed. I have hence for a long time trusted entirely to leeches and blisters, and have in almost every case, been in the practice of limiting the time the leeches were to be permitted to bleed, from so often witnessing the ill effects of such a drain on an already weakened frame."

As a very general rule as to the treatment, we agree with him. Our practice, in mild cases, consists in laxatives of oil, sponging with tepid water, diluents, occasionally diaphoretics or saline mixtures—although their good effects are often doubtful—and local bleeding or blisters when the head is much affected; stimulating pediluvia and sinapisms are also useful. At the close of the fever we give wine and a nourishing diet. In more severe cases, where there is extreme exhaustion, wine and analogous stimulants are necessary throughout the case, and the local derivatives are also of benefit. Sponging with solutions of chlorine, or vinegar and water, and free ventilation diminish the fœtor, and, by removing the accumulated exhalations, appear of decided benefit.

---

*On the use of Amadou as a surgical application.* By J. Wetherfield.—This substance, in popular use for lighting pipes, is not unknown to the profession, having been recommended as a remedy to suppress hæmorrhage; but I am not aware that it has ever been applied to any other purpose. From its extremely soft and elastic nature, this substance is decidedly superior to every other at present in use, both as a medium for applying support and pressure, and for defending the tender and inflamed skin, arising either from friction or the pressure of hard bodies; and I have accordingly for several years employed it with success in the form of a graduated compress in the umbilical hernia of new-born infants, and as a compress over fistulous ulcers, as in the groin, after suppurating bubo, instead of the usual mode of a graduated compress of lint, which soon loses its elasticity, and becomes a source of irritation over the inflamed skin, especially if the patient is obliged to use exercise. A graduated compress of amadou, applied in conjunction with the spica bandage, will be found to facilitate the healing of these fistulous ulcers. In defending parts from pressure it is an admirable application, preventing those ulcerations so often produced by long confinement to one position; for this purpose it may be spread with soap plaster, and applied over the sacrum and hips; and it is equally efficient as a defence against the galling occasioned by the springs of steel trusses in persons of spare habit, and whose skins are irritable. It forms the best corn plaster I have ever used, so shaped as to take off the pressure from the central prominent and painful thickened cuticle, and spread with a mild and anodyne plaster, it effectually defends them, relieves the pain, and they are removed by the process of absorption: as a support to varicose veins, it has answered better than rollers, laced stockings, or any of the contrivances at present usually employed for that purpose, giving a steady, equable support without the aid of a roller, the substance and elasticity of the material itself affording sufficient resistance to the enlarged veins; for this purpose it should be spread with soap plaster, and applied, if possible, in one piece over the limb. Repeated trials have proved the efficacy of the amadou in the cases above mentioned; and by directing the attention of the profession to it, other uses will soon be found to which it may be applied with advantage: the manufacturers, too, will be more careful to select the purer sorts of it for medical purposes, as at present it is rather difficult to obtain a supply sufficiently equable in surface and substance to apply with success in cases where a piece of considerable size is required, as for covering a limb for varicose veins.

We have employed the article here recommendad many years ago, at whose suggestion we do not recollect, but it certainly furnishes an admirable compress, under many circumstances, when used beneath a roller. The chief objection to it consists in the difficulty of procuring the article in sufficiently large quantities at a reasonable price.

To its employment in umbilical hernia we should decidedly object, being fully convinced, from very ample observation, as well as from the application of just mechanical principles, that, *at any age*, a spring truss, properly formed, will retain an umbilical hernia with much more certainty, and much less force than any other contrivance; that the contact of a smooth hard-wood block is less irritating to the skin than any soft compress, if the former be of the proper shape; and that the pressure of a circular bandage or strap around the abdomen, in order to confine a compress, is unphilosophical and inefficient, liable to produce protrusion elsewhere, necessarily more violent in its action than the spring, embarrassing to the growth of abdominal organs, and in every respect injudicious.

---

*Statistics of the Lateral Operation of Lithotomy at Naples.* By M. S. de Renzi.—From the year 1821 to 1828, inclusive, 596 cases of stone were operated on, 579 of whom were males, and only 17 females. Of this number 503 recovered, and 91 died; 306 of these cases occurred in persons under 15 years of age; 231 in adults, and 59 in aged individuals. As a means of comparing the mortality from this operation, the results of the operations of lithotomy for the year 1839

are given. During this year 47 were operated on, 46 of whom were men, and 1 woman. Of these 38 recovered, and 9 died. Of this number 15 were below 15 years of age; 31 were adults, and 1 was aged.—*Ed. Med. and Surg. Journ., from Il Filiatre Sebezio Giornale.*

---

*Reduction of a Strangulated Hernia, apparently effected by Acupuncture.* By Dr. Daser.—A man, 50 years of age, was seized with strangulated hernia, with vomiting of stercoral matter, and all the other usual symptoms. The taxis having failed, Dr. Daser, before having recourse to the operation for strangulated hernia, with the view of trying the effect of acupuncture, for the purpose of evacuating the gaseous contents of the strangulated portion of intestine, made two punctures in it with a long fine needle. No gaseous matters apparently escaped, but the patient complained of acute pain, and loud gurgling sounds were heard in the abdomen, immediately after which the hernia was spontaneously reduced. Dr. Daser attributed this fortunate occurrence to the prick of the needle having excited contractibility of the intestine, causing it to contract on its contents, expel the gaseous matter into the abdominal portions of the gut, and thus facilitate its reduction.—*Ibid., from Journal fur Chirurgie und Augenhetlkunke,* 1840.

---

*Hæmorrhage after Lithotomy stopped by Creosote.* By Dr. Daser.—In a case of lithotomy it was found impossible to arrest the hæmorrhage by any of the usual means, and no particular vessel could be discovered from which the blood might flow. The patient was at last reduced to the lowest ebb from the continued loss, and had already lost consciousness, when a sponge dipped in pure creosote was introduced into the wound, and pressed against the bleeding parts for an instant or two. The hæmorrhage was immediately arrested. No particular pain was experienced, no unpleasant symptoms followed: thin eschars were thrown off, and the patient recovered.—*Ibid., from Journ. fur Chir. und Aug.,* 1840.

---

*The conditions which favour an excessive size of the Fœtus.*—Professor Osiander regards a too exclusive use for food, of articles composed of fecula, especially rye-bread, as contributing to produce a sort of fœtal hypertrophy; and he recommends to pregnant women who are obliged to live on such a diet, to abstain at regular times from food, and to take occasionally a saline purgative. The excessive developement of the fœtus is one of the causes of difficult labour, and the Professor relates five cases of this nature.—*American Journal of the Med. Sciences, from Zeitschrift fur gesammte Med.*

---

*The Medical Examiner* is published every Saturday by *J. C. Turnpenny*, N. E. corner of Spruce and Tenth streets. Terms—*three dollars* a year for one copy, or *five dollars* for two copies sent to the same post office, in all cases in advance. Postage the same as on newspapers. Letters to be addressed,—post-paid,—to the Editors of the Medical Examiner, Philadelphia.
*Reynell Coates, M. D.*, Acting Editor.
*J. B. Biddle, M. D.*, and *W. W. Gerhard, M. D.*, Co-editors.
*J. B. Biddle, M. D.* Proprietor.

